# Mitochondria engage the integrated stress response to promote tumor growth

**DOI:** 10.18632/oncotarget.28372

**Published:** 2023-02-25

**Authors:** Dillon P. Boulton, M. Cecilia Caino

**Keywords:** mitochondria, prostate cancer, integrated stress response

Prostate cancer (PCa) is the most diagnosed and second deadliest cancer among men in the United States, with an estimated 268,490 new cases and 34,500 deaths in 2022 (ACS Cancer Facts and Figures 2022). While the prognosis for men with early-stage disease remains extremely favorable (>99% 5-year overall survival, OS), men diagnosed with metastatic PCa have a 30% 5-year OS, clearly demonstrating a need for therapeutic options for these patients (ACS Cancer Facts and Figures 2022). Due to a strong reliance on androgens to drive PCa, first and second courses of therapy involve androgen deprivation therapy or targeting the androgen receptor directly in combination with several other cytotoxic agents [1–3]. Unfortunately, some tumors develop resistance to these androgen axis therapies and progress to castrate-resistant and metastatic PCa, which drives the majority of PCa deaths [3, 4]. This underscores a strong need to identify and characterize actionable targets within these tumors.

Of interest, mitochondria are emerging as critical organelles that promote tumorigenesis and metastasis [5]. Mitochondria promote tumor progression through their pleiotropic functions in ATP production, biosynthesis, calcium and iron homeostasis, and redox balance [6–10]. Along this line, we have recently described a novel signaling pathway where mitochondria promote castrate resistant metastatic PCa growth by acting as a signaling platform to facilitate efficient stress signaling [11]. This pathway is centered around mitochondrial Rho GTPase 2 (MIRO2), an outer-mitochondrial membrane protein in the Ras superfamily of GTPases [12, 13]. MIRO2, alongside its paralog MIRO1, were initially described in neurons for their function in linking mitochondria to kinesin and dynein motors to support efficient mitochondrial trafficking [13–16]. We and others have found that MIRO2 is dispensable for long distance mitochondrial transport in non-neuronal cells suggesting alternate functions in these cells [11, 17]. While an abundance of work has shown the importance of MIRO2 in many mitochondrial functions including actin-based mitochondrial movement, mitophagy, maintaining mitochondrial-endoplasmic reticulum contacts, and proper formation of cristae morphology in non-tumorigenic cells, the function of this protein in the context of cancer was virtually unknown [17–20].

Our work identified that patients with recurred/progressed disease had higher MIRO2 mRNA expression and higher MIRO2 expression correlated with worse disease-free survival for these patients. Rigorously using cell models representing androgen sensitive and androgen insensitive disease, we found that MIRO2 was universally critical for PCa cell growth and survival. Importantly, this was replicated in xenograft *in vivo* models, with MIRO2 expression strongly correlating with growth of PCa tumors. To determine how MIRO2 supported PCa growth and survival, we identified novel MIRO2 binding partners through co-immunoprecipitation mass spectrometry. We explored the top hit from our screen, General Control of Non-derepressible (GCN1), and found that GCN1 was also crucial for the growth and survival of PCa cells. GCN1 acts as the upstream activator of GCN2—one of the major kinases involved in the integrated stress response (ISR)—which functions to sense a variety of intracellular stresses including amino acid availability, redox stress, and actin dynamics cues [21, 22]. Interestingly, ablation of MIRO2 significantly dampened the cells ability to activate the ISR in response to amino acid deprivation. This included activation of GCN2, activation of eIF2α, and translation of ATF4. Furthermore, the effect of MIRO2 on PCa cell growth was primarily mediated by ATF4 expression.

This study adds to an ever-growing body of data that highlights the importance of mitochondria—outside their traditional roles as metabolic hubs—as important signaling nodes to promote tumor growth. It is interesting to note that ablation of MIRO1 had no effect on PCa growth, survival, or stress signaling in response to nutrient deprivation, providing solid evidence of non-overlapping functions in these contexts. This is in contrast to recent studies performed in mouse embryonic fibroblasts, which require loss of both MIRO1 and MIRO2 to affect mitochondrial functions, which suggests that MIRO1 and MIRO2 are functionally redundant or cooperate to maintain cellular health [17, 20]. Whether the ability for MIRO1 to compensate for MIRO2 is lost in cancer cells or if the ability to compensate is dependent on the function requires further investigation.

Another major discovery was the identification of many previously unknown binding partners for MIRO2. Interestingly, many canonical MIRO2 interactors that have been characterized in non-tumorigenic cells, were not found in our screen. This gives rise to the possibilities that the function of MIRO2 could be completely different in 1) cancer vs. normal cells or 2) stressed vs. non-stressed cells. Furthermore, while the interaction of MIRO2 and GCN1 was readily found in many PCa cell lines and tumor samples from patients, this interaction was almost completely lost in normal immortalized cell lines or normal prostate epithelia from patient samples. This suggests that targeting the MIRO2-GCN1 axis would have limited off target effects towards normal cells in patients.

Our research also found that solid tumors established in mice strongly induced the activation of GCN2. This is consistent with new research studies that also show GCN2 to be a critical sensor of mitochondrial health and for PCa tumor growth [23, 24]. Excitingly, two GCN2 kinase inhibitors have been developed and tested *in vivo* [25, 26]. While GCN2 inhibitors alone have shown no efficacy using *in vivo* models of acute lymphocytic leukemia, strong anti-proliferative effects have been observed when used in combination with asparaginase (ASNase)-dependent depletion of asparagine and glutamine in serum [25, 26]. In this context, treatment with ASNase forced cells to activate GCN2 to survive. It is interesting to speculate that in solid tumors that activate GCN2 without ASNase—like the ones formed in our *in vivo* xenografts tumors—treatment with GCN2 inhibitors alone may be enough to induce anti-proliferative effects.

Overall, this research proposes a new paradigm on how AR-independent PCa can drive tumor traits by exploiting mitochondrial signaling pathways and offers a fresh opportunity to develop novel targeted therapies for patients with prostate cancer.
